# Supplementary material for: Epilepsy and intellectual disability linked protein Shrm4 interaction with GABABRs shapes inhibitory neurotransmission
Source: Nat Commun. 2017 Mar 6;8:14536. doi: 10.1038/ncomms14536 (PMC5343488; doi:10.1038/ncomms14536)
Supplement: Supplementary Information — Supplementary Figures and Supplementary Tables [file ncomms14536-s1.pdf]

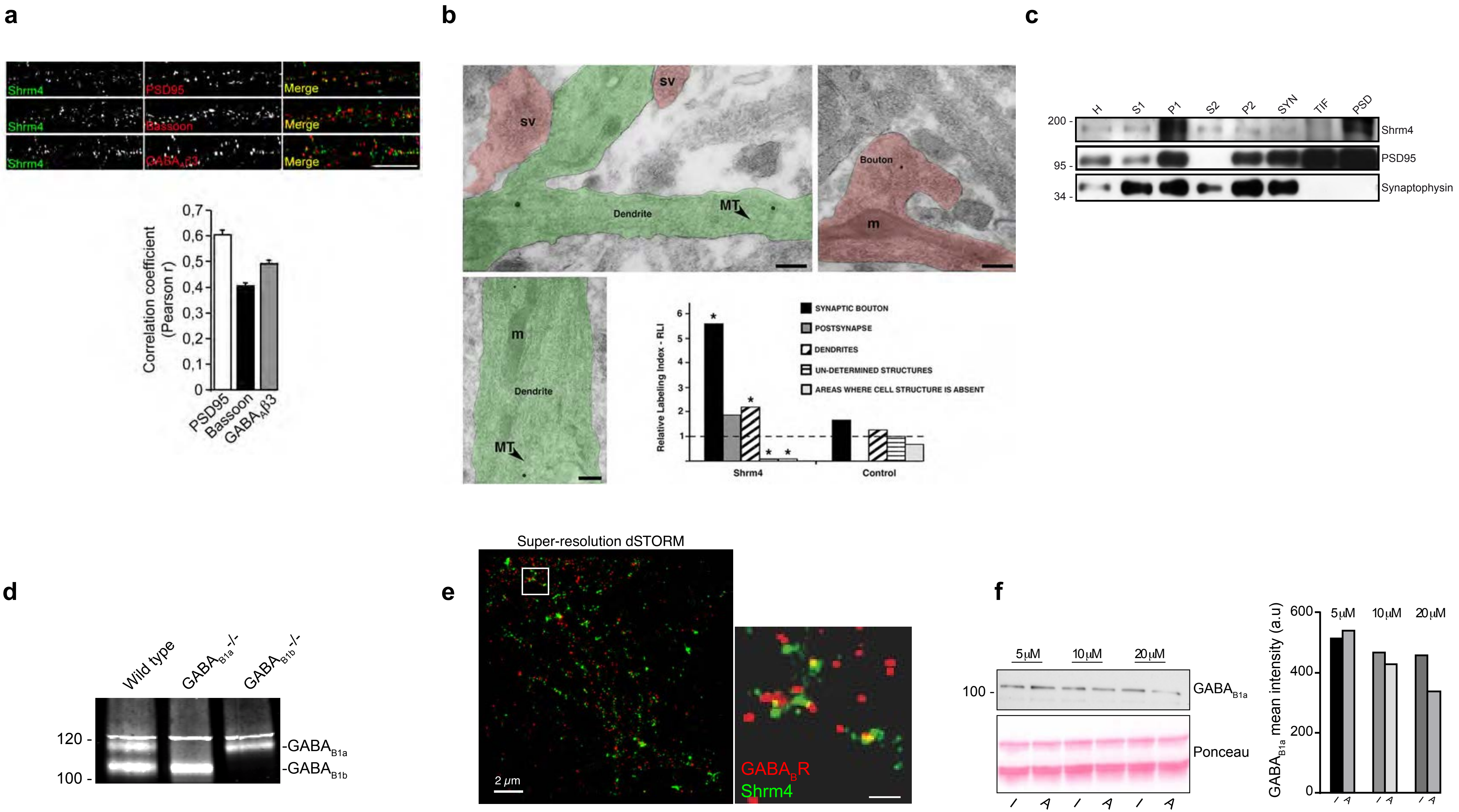

Supplementary Figure 1

Supplementary Figure 1. Shrm4 is localized in the pre- and postsynaptic compartments of neurons.

(**a**) (Top) Images and (Bottom) histogram showing quantitative co-localization of endogenous Shrm4 staining with presynaptic (Bassoon) as well as with postsynaptic markers of both excitatory (PSD-95) and inhibitory ($GABA_A\beta3$) synapses in 18*DIV* (days *in vitro*) cultured hippocampal neurons. n= 15, Scale bar: 10 μm (**b**) Electron micrographs of cultured cortical neurons at 20*DIV* immunolabeled with anti-Shrm4 and secondary antibody conjugated with Nanogold particles, showing gold particles in dendrites and synaptic boutons. Sv: synaptic vesicles; MT: microtubules; m: mitochondria. Scale bar: 200 nm. The histograms show that in Shrm4-stained neurons, gold particles were significantly enriched (*p <0.0001, see Table 1) in dendrites and presynaptic terminals (synaptic boutons) and also tended to accumulate at the postsynaptic compartments. (**c**) Western blot analyses of postsynaptic density (PSD) subcellular fractionation from rat brain. H, total homogenate; S1, P1, Whole-brain postnuclear supernatant and membrane fractions, respectively; S2, cytosolic proteins; P2, crude synaptosomal fraction; SYN, synaptosomes; TIF, Triton X-100 insoluble fraction; PSD, Triton X-100-extracted PSD. (**d**) Western blot of wild-type, $GABA_{B1a}^{-/-}$ and $GABA_{B1b}^{-/-}$ mouse brain lysates using anti-$GABA_{B1}$ for immunoblotting. The blot shows that anti-$GABA_{B1}$ antibody recognizes both $GABA_{B1a}$ and $GABA_{B1b}$ isoforms. (**e**) Full image of direct stochastic optical reconstruction microscopy (dSTORM) imaging of $GABA_B$R-ATTO 488 (shown in red) and Shrm4-Alexa 647 (shown in green) on *14DIV* cultured hippocampal neurons. Scale bar: 0.4 μm. (**f**) (Left) GST pull-down experiments using GST-PDZ on lysates of cells overexpressing $GABA_{B1a}$-GFP with increasing concentrations (5, 10 and 20 μM) of Tat-control (I) or Tat-859-870 (A) peptide. (Right) Histogram showing the $GABA_{B1a}$ mean intensity compared to the Tat-control peptide. Incubation with the Tat 859-870 at 10 and 20 μM showed a reduction in Shrm4/$GABA_{B1}$ binding compared to the Tat control peptide.

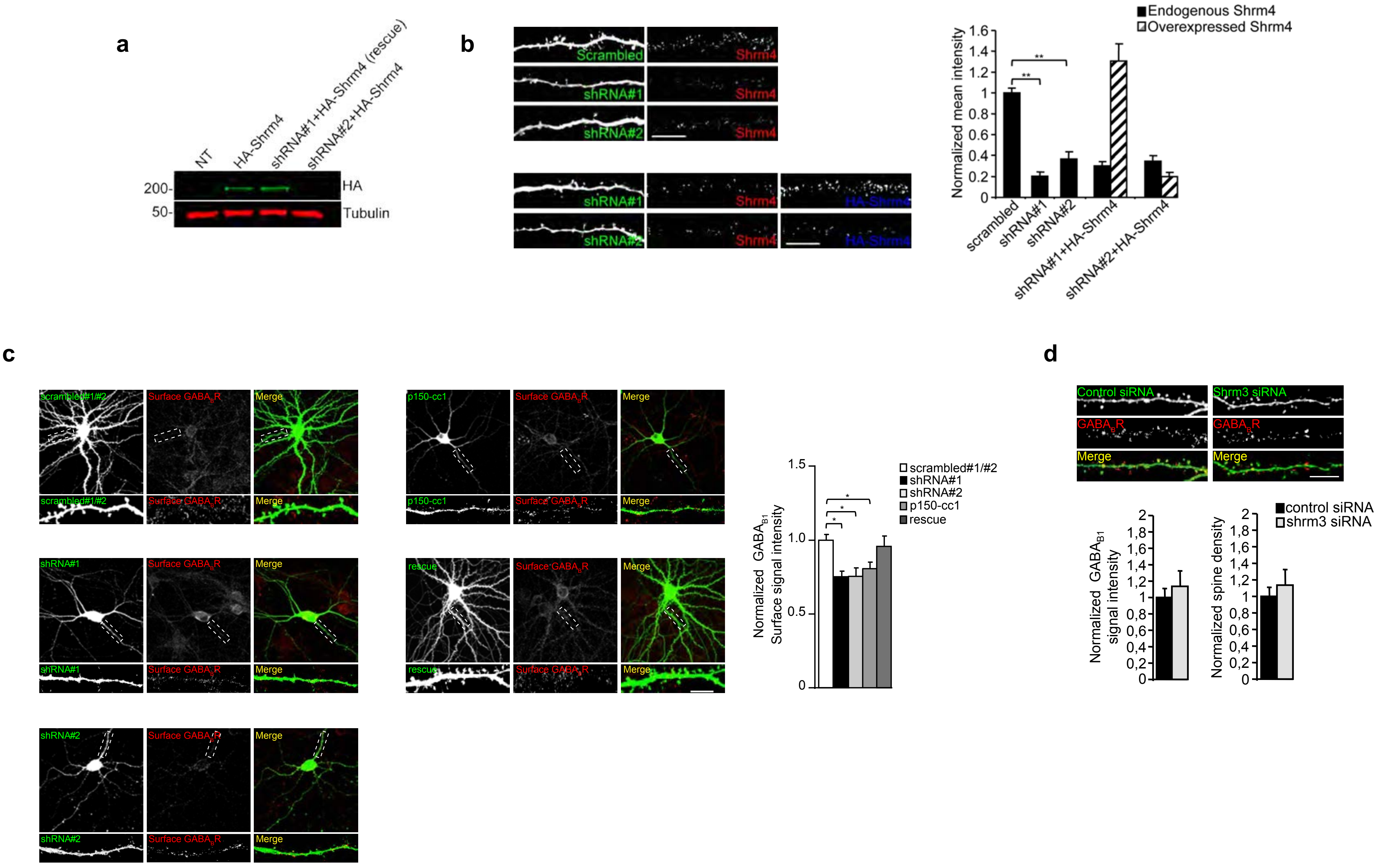

Supplementary Figure 2

Supplementary Figure 2. Validation of knockdown and rescue constructs.

(**a**) Western blots of lysates from transfected and non-transfected (NT) HEK293 cells demonstrating the effectiveness of the two knockdown constructs (shRNA#1 and shRNA#2). HA-tagged Shrm4 (HA-Shrm4) was used to overexpress Shrm4 in these cells. Transfected constructs were HA-Shrm4, shRNA#1 with HA-Shrm4 (rescue) or HA-Shrm4 with shRNA#2. α-tubulin served as loading reference and polyclonal anti-HA antibody was used to reveal Shrm4 expression. The blot shows that HA-Shrm4 is resistant to shRNA#1 but not to shRNA#2. (**b**) (Left) Representative images of hippocampal neurons transfected at 8*DIV* and immunostained at 18*DIV* with polyclonal anti-Shrm4 antibody to reveal endogenous Shrm4 expression and monoclonal anti-HA to reveal overexpressed Shrm4. Transfections were scrambled, shRNA#1, shRNA#2, shRNA#1 plus HA-Shrm4, and shRNA#2 plus HA-Shrm4. Scale bar: 10 μm. (Right) Quantification of dendritic Shrm4 expression normalized to scrambled transfected neurons. Both shRNA#1 (**$p < 0.01$; one-way ANOVA, $n = 10$) and shRNA#2 (**$p < 0.01$; one-way ANOVA, $n = 10$) reduces endogenous Shrm4 expression and expression of HA-Shrm4 (resistant) with shRNA#1 rescues Shrm4 expression whereas it does not with shRNA#2. (**c**) Full representative images and histogram showing surface immunostaining for $GABA_BR$ in cultured hippocampal neurons at 18*DIV* after transfection with scrambled, shRNA#1, shRNA#2, p150-cc1 or rescue constructs at 8*DIV*. Scale bar: 15 μm. Histograms show mean ± SEM; $n = 5\text{-}15$, *$P < 0.05$, One-way ANOVA. (**d**) Representative images of hippocampal neurons transfected at 8*DIV* with GFP coexpressing-control siRNA or Shrm3 siRNA and immunostained at 18*DIV* for surface $GABA_{B1}$. No significant changes in $GABA_{B1}$ intensity or spine density were observed from Shrm3-siRNA expressing neurons compared to controls. Histograms show mean ± SEM; $n = 10$.

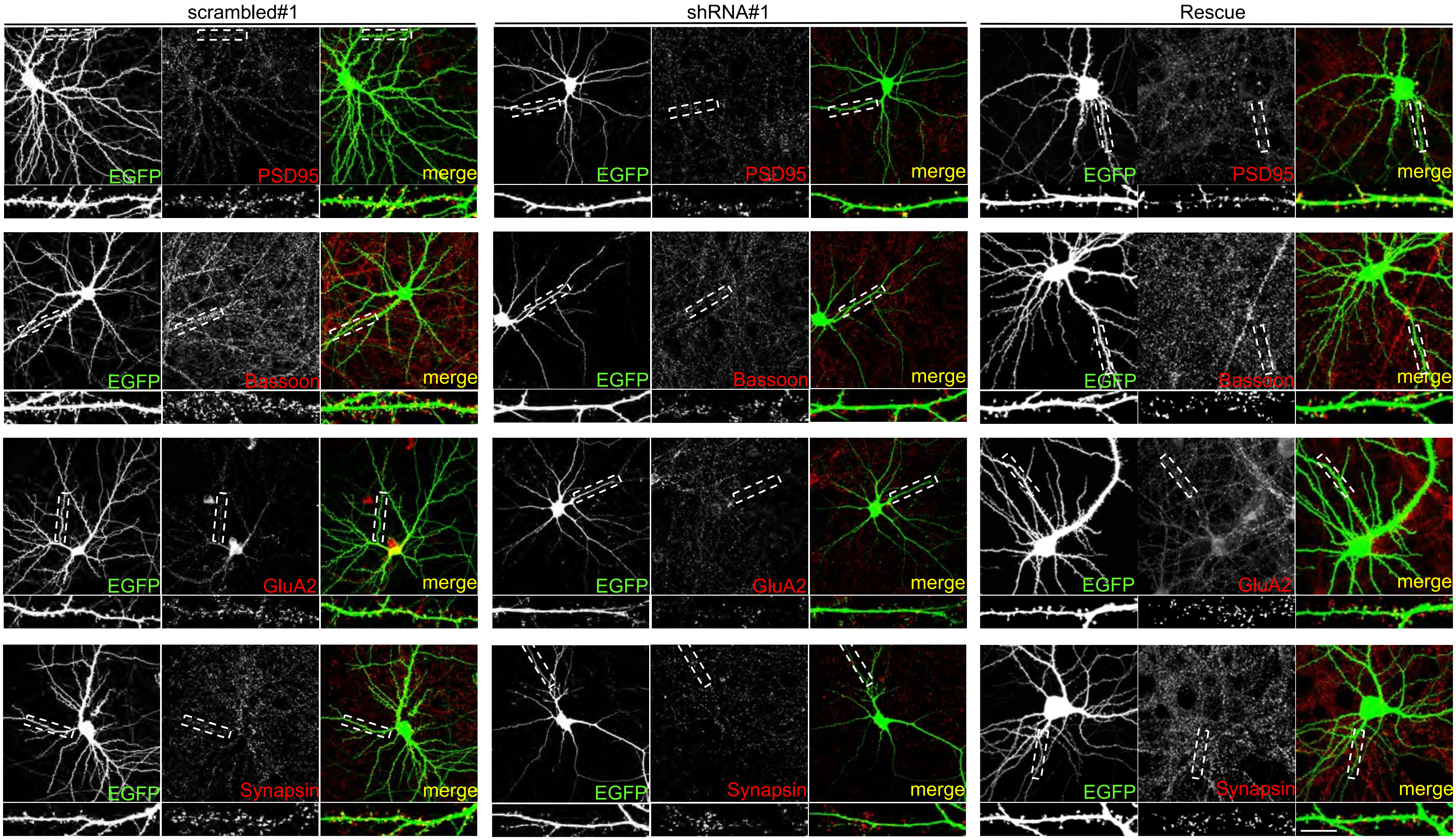

Supplementary Figure 3

Supplementary Figure 3. Shrm4 regulates synaptic protein composition.

Full representative images of rat hippocampal neurons transfected at 8*DIV* with Shrm4 scrambled#1 or shRNA#1 with or without rescue constructs and immunostained for PSD95, Bassoon, GluA2 and Synapsin post-synaptogenesis at 18*DIV*. Scale bars: 10 µm.

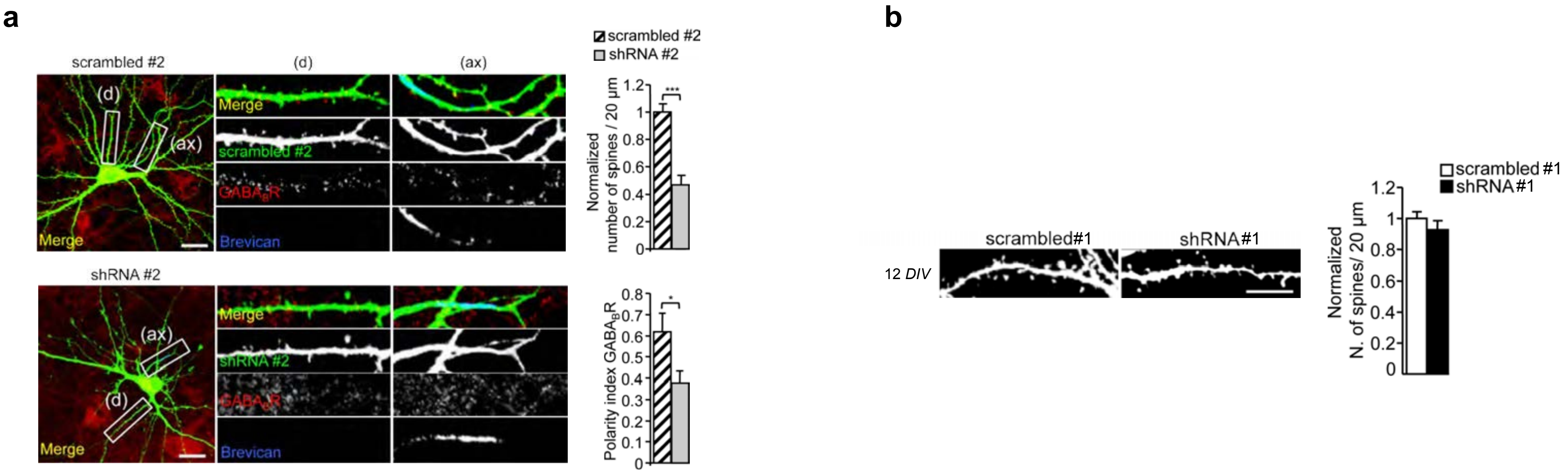

Supplementary Figure 4

Supplementary Figure 4. Shrm4 regulates dendritic spine density and GABA$_B$R transport before synaptogenesis.

(**a**) Representative images and histograms of dendrites (d) and axons (ax) at 1*4DIV* from hippocampal neurons transfected before synaptogenesis at 8*DIV* with control (scrambled#2) or knockdown (shRNA#2) shRNAs. The dendritic spine density and GABA$_B$R expression levels were measured in dendrites and axon by co-staining for brevican to localize axons. The histograms compare mean (± SEM) spine density (normalized number of spines/20 μm; 20 neurons examined per construct) and polarity index (PI) of GABA$_B$R expression. Spine density (***$p < 0.001$; t-test, $n = 15$) is significantly lower in Shrm4-silenced neurons than scrambled controls. In addition, the PI of Shrm4 knockdown neurons is significantly lower than scrambled (*$p < 0.05$; t-test, $n = 10$). Scale bar: 20 μm. (**b**) Representative images with histograms comparing spine density of rat hippocampal neurons transfected at 12*DIV* with knockdown shRNA or scrambled shRNA. Spine density does not differ between Shrm4-silenced and control neurons when transfected post synaptogenesis at 12*DIV* (normalized number of spines/ 20 μm) Histogram mean ± SEM, n=20.

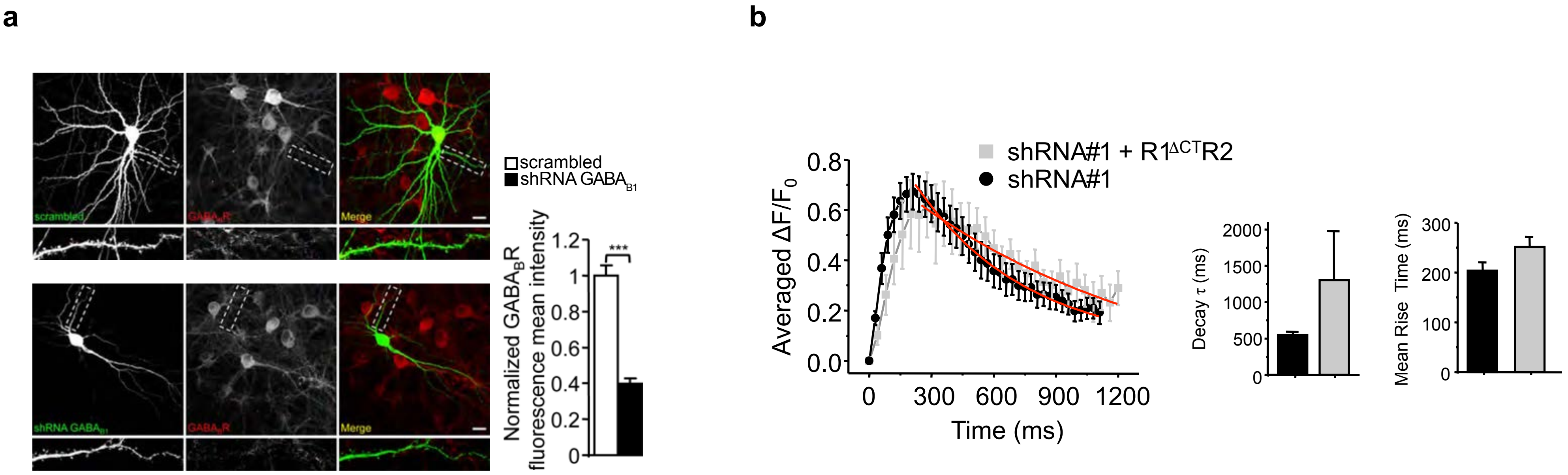

Supplementary Figure 5

Supplementary Figure 5.

(a) Validation of $GABA_{B1}$ shRNA. Representative images (Left) and histogram (Right) of hippocampal neurons transfected at 8*DIV* with scrambled (control) or knockdown shRNA that specifically target $GABA_{B1}$. The neurons were transfected at 8*DIV* and analyzed by confocal microscopy at 18*DIV* for $GABA_BR$ expression. Normalized $GABA_BR$ fluorescence intensity is significantly lower in shRNA-transfected neurons than scrambled controls. The histograms compare mean (±SEM); n=20; ***$p < 0.001$, t-test. Scale bar: 20 μm.

(**b**) **Calcium dynamics in neurons expressing R1ΔCT.** (Left) Time course of averaged $\Delta F/F_0$ values and single exponential fits of Ca^{2+} signals in control neurons expressing shRNA#1 and neurons expressing R1b subunits with a deletion of the C-terminus tail (R1ΔCT) co-expressed with R2 and shRNA#1. Red lines show exponential fits of the decays. (Right) Mean rise times and decay τ of Ca^{2+} signals for control and R1ΔCT. Histograms show mean ±SEM; n = 11-18.

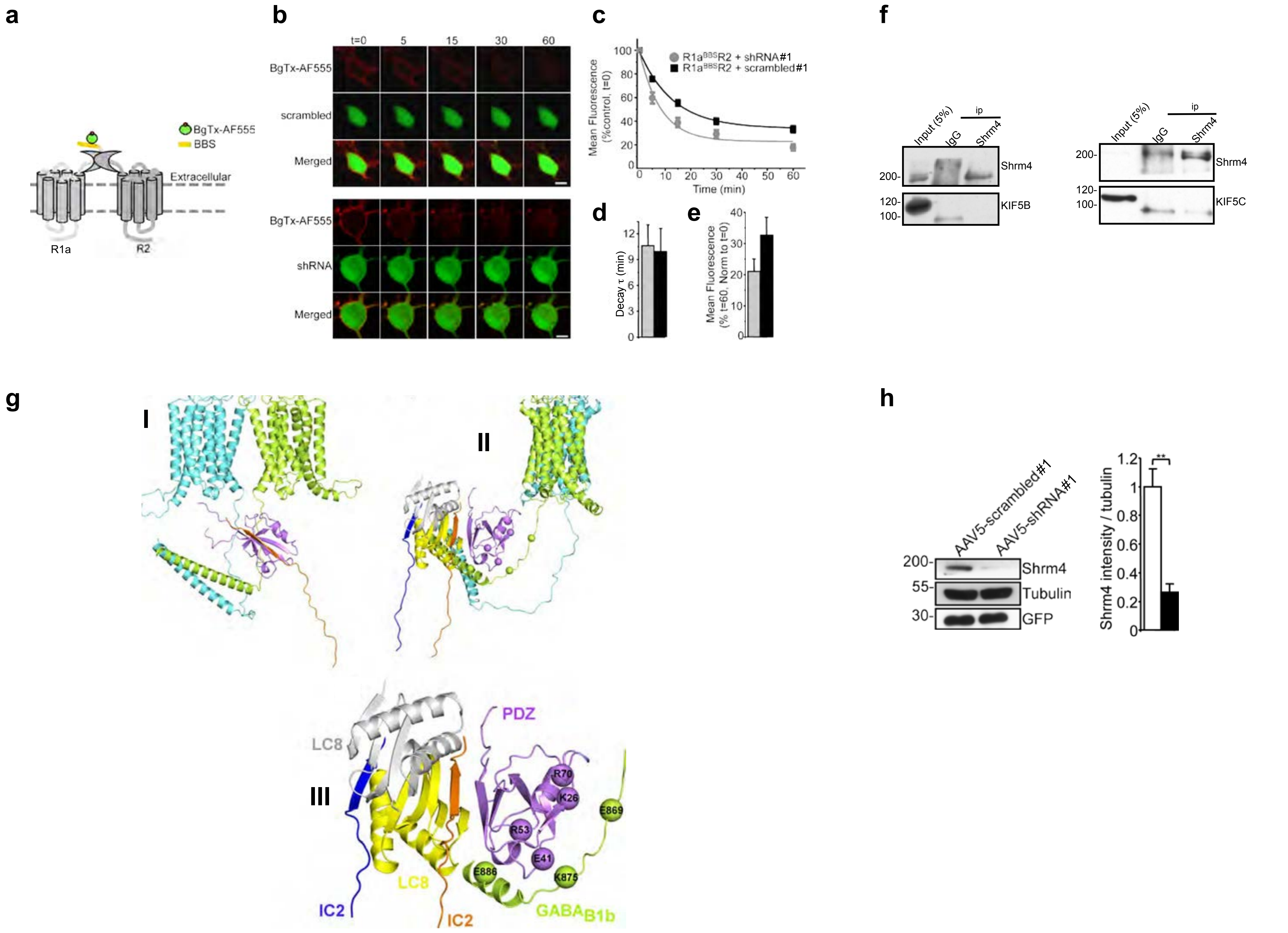

Supplementary Figure 6

Supplementary Figure 6. Shrm4 and dynein/dynactin regulate $GABA_BR$ transport to dendrites.

(**a**) Schematic showing a $GABA_BR$ heterodimer with an α-bungarotoxin (BgTx) binding site (BBS) in the N-terminus of R1a ($R1a^{BBS}$). (**b**) Live cell surface fluorescence monitored over 60 min at 30-32°C, in hippocampal neurons at 14*DIV*, expressing $R1a^{BBS}R2$ with BgTx coupled to Alexa-fluor 555 (BgTx-AF555), with either Shrm4 knockdown (shRNA#1) or scrambled constructs (scrambled#1). (**c**) Rates of constitutive internalization for BgTx-AF555-tagged $GABA_BRs$ in Shrm4 knockdown (●) and scrambled controls (■). (**d**) Exponential decay time constants for membrane fluorescence for $GABA_BRs$ in knockdown and control cells. (**e**) Membrane fluorescence of $GABA_BRs$ after 1 hr in knockdown and scrambled control cells. Histograms show mean ±SEM; n = 6-8. Scale bar 10 µm. (**f**) Blots of Shrm4 immunoprecipitation experiments on rat brain lysates, which demonstrate the absence of interaction with the subunit B and C of KIF5. (**g**) Supramolecular complex between the Shrm4 PDZ domain, $GABA_{B1b}$, $GABA_{B2}$ and the 104-138 filaments of dynein IC2 interacting with dimeric dynein LC8. (I) The $GABA_{B1b}$ (green) and $GABA_{B2}$ (aquamarine) modeled heterodimer comprises the seven-helices bundle, the intracellular and extracellular loops, and the C-terminus up to the end of the coil-coil region. The Shrm4 PDZ is violet. The 110-138 filament of dynein IC2C is orange. (II) A side view of the complex in (I) is shown. In this panel dimeric LC8 (gray and yellow protomers) and an additional filament of IC2C (blue) of dynein are shown as well. The spheres centered on the Cα-atoms of $GABA_{B1b}$ and PDZ mark a selection of salt bridges at the interface between the two proteins. (III) A zoom on the $GABA_{B1b}$/PDZ/IC2/LC8 interfaces is shown. The C-terminal 867-886 amino acid segment of $GABA_{B1b}$ makes contacts with PDZ. The selected salt bridges marked by spheres at the $GABA_{B1b}$-PDZ interface include: 1) E869 from the receptor and both K26 and R70 from β2 and α2 of PDZ, respectively; 2) K875 from the receptor and E41 from the β2-α1 loop of PDZ;

and 3) E886 from the coil-coil helix of the receptor and R53 from the α1-β4 loop of PDZ. The β1 strand of PDZ forms an antiparallel β-sheet with the 128-135 strand of IC2 (orange). The latter in turn forms an antiparallel β-sheet with the yellow protomer of LC8. A second segment of IC2 (blue) forms an antiparallel β-sheet with the gray protomer of LC8. For both IC2C filaments of dynein the 110-120 segment is not shown. (**h**) Representative Western blots of Shrm4, α-tubulin and GFP from hippocampal brain extracts prepared three weeks after injection of AAV5-scrambled#1 or AAV5-shRNA#1 (knockdown) into opposing hippocampi of rats. Hippocampi infected with AAV5-shRNA#1 show robust reduction in Shrm4 expression (Histograms show mean ±SEM; $n = 3$; $^{**}p < 0.01$; t-test).

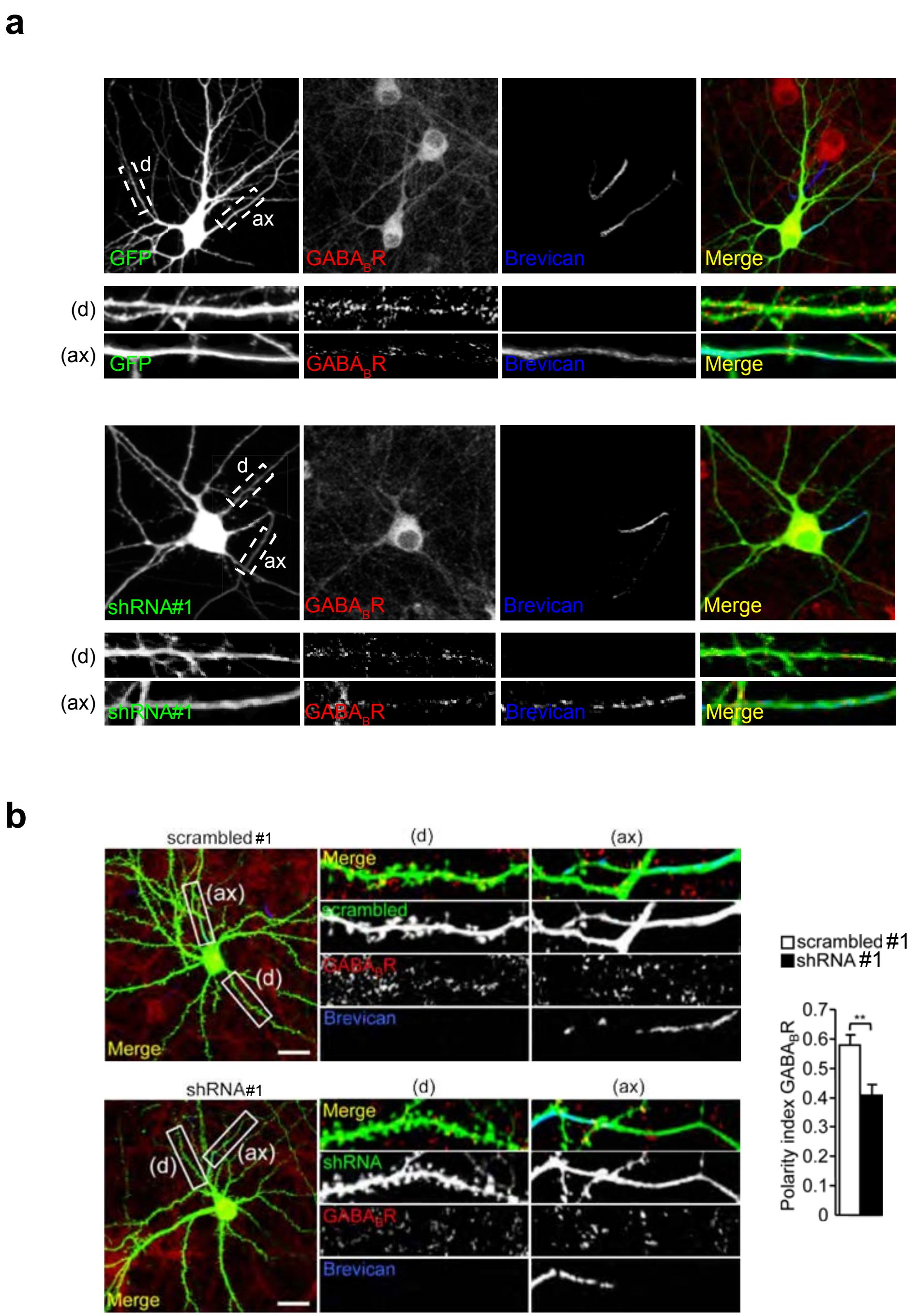

Supplementary Figure 7

Supplementary Figure 7. Shrm4 and dynein/dynactin regulate GABA$_B$R transport to dendrites.

(a) Full representative images of immunostaining of endogenous GABA$_B$R on dendrites (d) and axons (ax) in 18*DIV* neurons transfected at 7*DIV* with Scrambled, shRNA#1, Rescue or p150-cc1 construct. Scale bar 10 µm. **(b)** Representative images of dendrites (d) and axons (ax) of 18*DIV* hippocampal neurons transfected after synaptogenesis at 12DIV with knockdown or scrambled controls. Neurons were immunostained to reveal endogenous GABA$_B$Rs and brevican to localize axons. Histogram showing that PI of knockdown shRNA-expressing neurons is lower than that of scrambled-expressing neurons (Histograms show mean ±SEM; n = 10; $**p < 0.01$; t-test,). Scale bar: 20 µm.

a

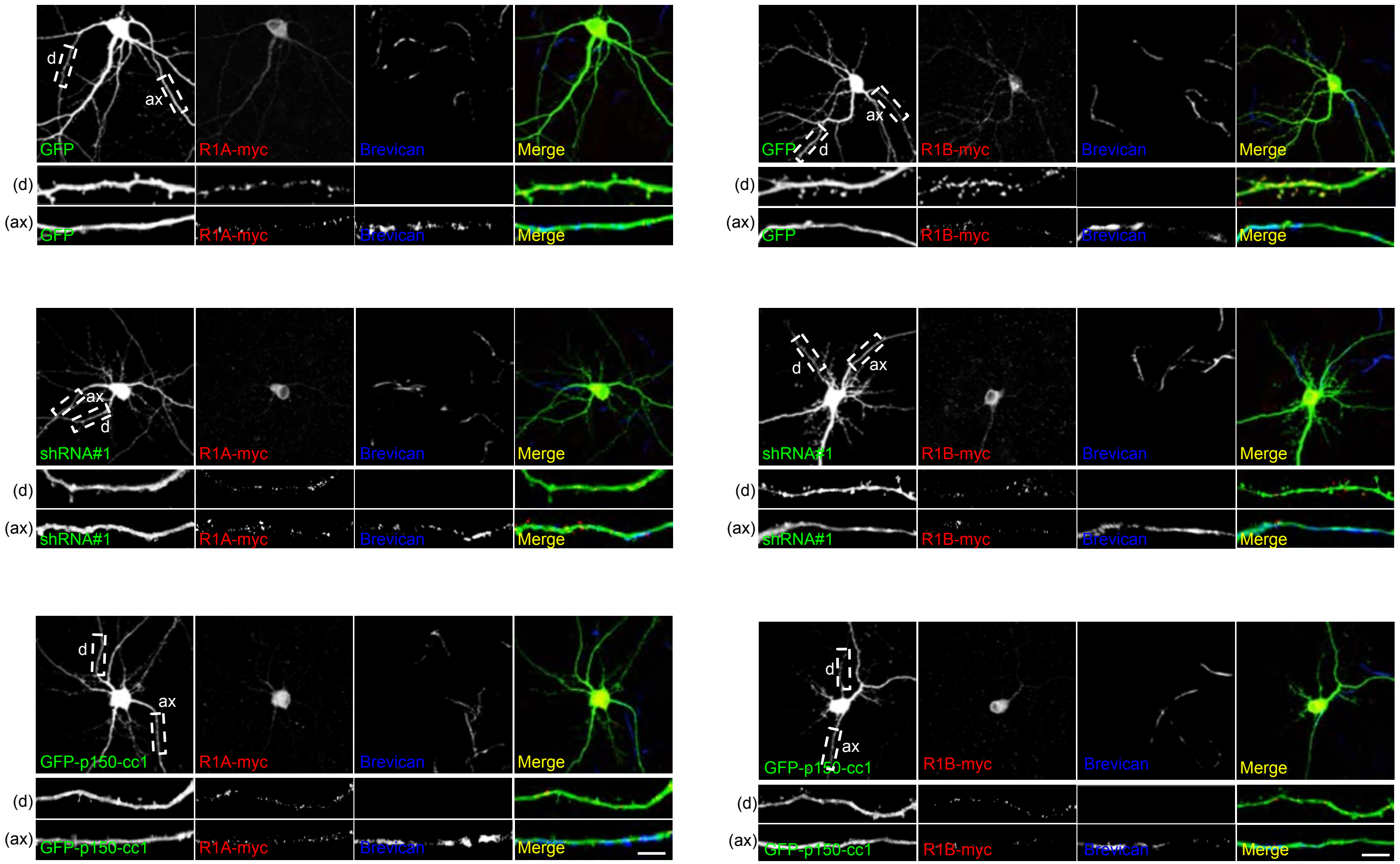

b

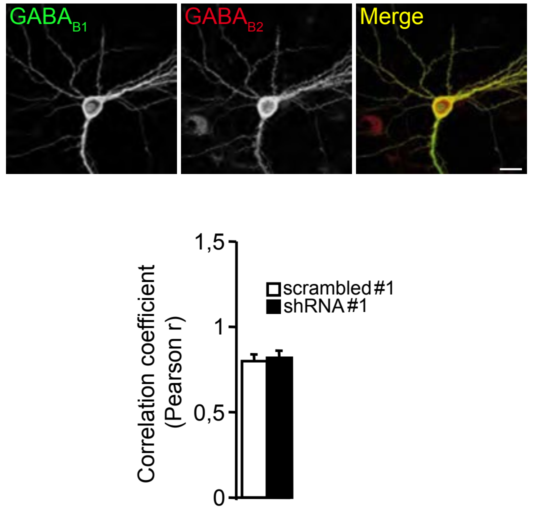

Supplementary Figure 8

Supplementary Figure 8. Shrm4 and dynein/dynactin regulate overexpressed $GABA_BR$ transport to dendrites.

(**a**) Full representative immunostaining images of overexpressed myc-tagged $GABA_BR$-1a (left) and -1b (right) on dendrites (d) and axons (ax) at 18*DIV* neurons, transfected at 7*DIV* together with $GABA_{B2}$ and either scrambled, shRNA#1, Rescue or p150-cc1 construct (**b**) Representative images (top) and histogram (bottom) of 18*DIV* cultured hippocampal neurons transfected at 8*DIV* with $GABA_{B1}$-myc and $GABA_{B2}$-flag with either knockdown shRNA#1 or its scrambled control and immunostained using the anti-tag antibodies. A high degree of co-localization is seen for both knockdown and scrambled shRNA-expressing neurons.

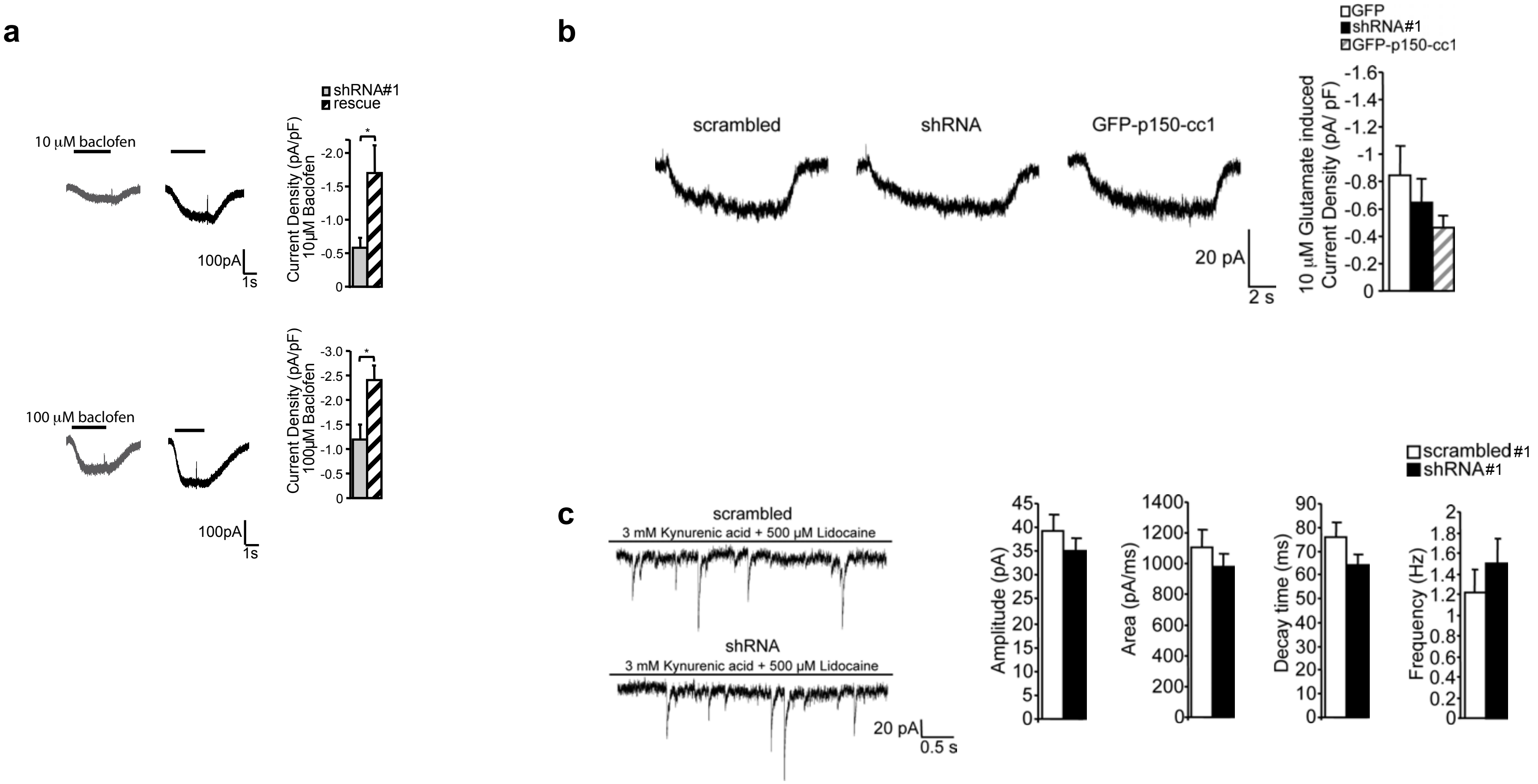

Supplementary Figure 9

Supplementary Figure 9. Changes in electrophysiological properties *in vitro* upon Shrm4 silencing.

(**a**) Whole-cell GIRK currents, recorded in response to $GABA_BR$ activation by 10 or 100 µM baclofen, from rat hippocampal neurons at 14*DIV*, transfected with knockdown shRNA#1 with or without the rescue construct (HA-Shrm4). Peak K^+ current density in Shrm4-silenced neurons was significantly lower than in the presence of the rescue construct for both doses of baclofen. Histograms show mean ±SEM; *P<0.05, t-test, n = 7-10. (**b**) Whole-cell GIRK currents recorded from cultured rat hippocampal neurons at 14*DIV* in response to glutamate do not differ between neurons expressing knockdown (shRNA#1), those expressing scrambled#1, or those expressing the coiled-coil domain of dynactin (GFP-p150-cc1). GIRK currents were induced by mGluR activation with 10 µM glutamate in the presence of 2 mM kynurenic acid and 20 µM bicuculline. (**c**) Amplitude, decay time, area and frequency of mIPSCs recorded from 14*DIV* hippocampal neurons transfected (8*DIV*) with knockdown shRNA#1 or scrambled#1, measured in the presence of kynurenic acid (3 mM) and lidocaine (500 µM) Histograms show mean ±SEM; n=12-17.

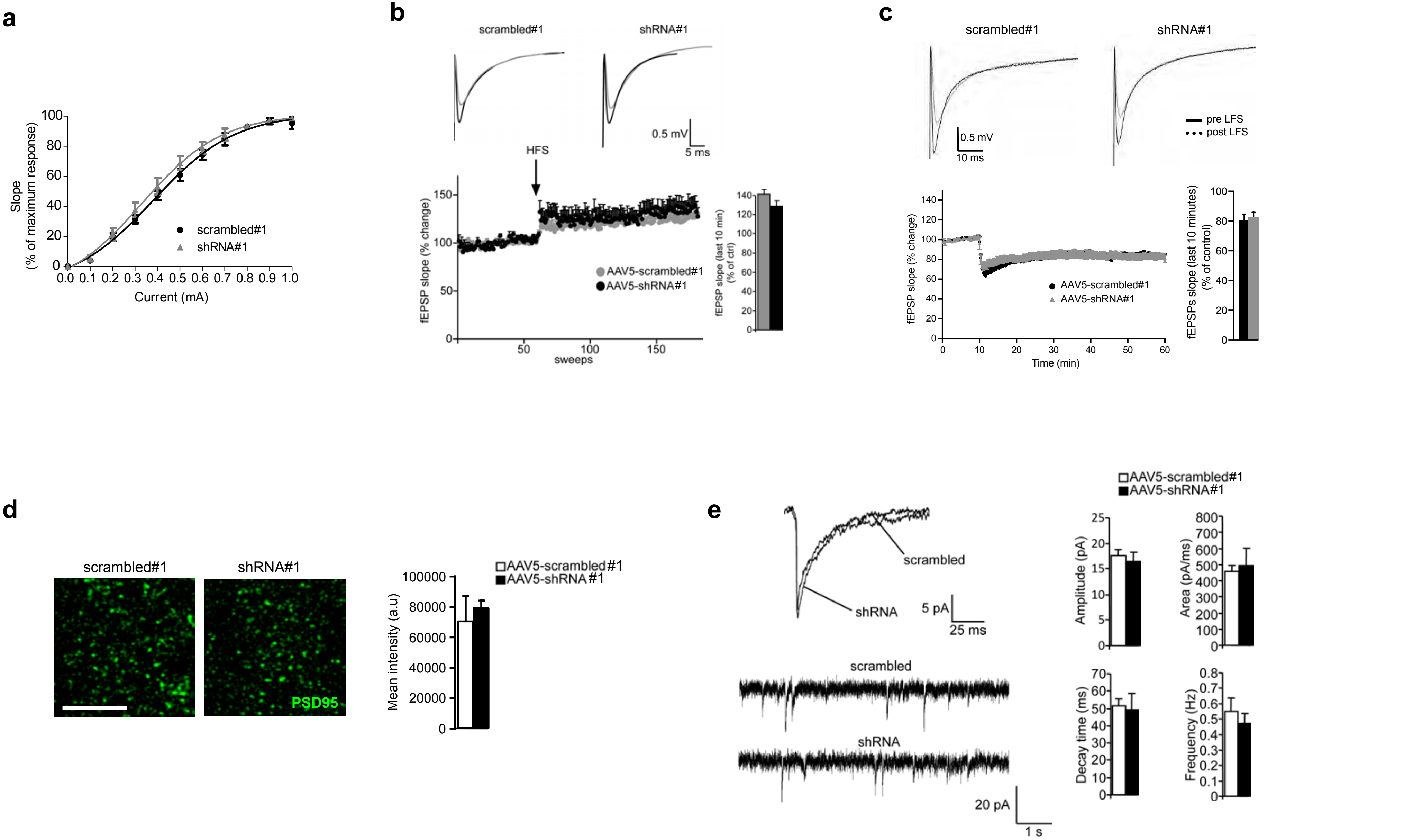

Supplementary Figure 10

Supplementary Figure 10. Changes in electrophysiological properties *in vivo* upon Shrm4 silencing.

(**a**) Input-output curves measured *in vitro* for field excitatory postsynaptic potential (fEPSP) in hippocampal slices from rat injected with AAV5-shRNA#1 compared to AAV5-scrambled#1 controls. n = 6 (**b**) Individual traces of field excitatory postsynaptic potentials (fEPSPs) at baseline (grey) and after high frequency stimulation (black) of acute hippocampal slices of adult rats previously injected with either knockdown AAV5-shRNA#1 (right hemisphere) or AAV5-scrambled#1 (left hemisphere) (n = 9 animals). (Bottom) Representative traces and bar chart of changes in fEPSPs. Baseline fEPSPs were recorded from the apical dendritic layer of CA1. LTP was induced by high frequency stimulation (100 stimuli at 250 Hz) of Schaffer collaterals. The traces show no differences in LTP induction or maintenance between Shrm4-silenced hippocampal slices and scrambled controls. (**c**) Individual representative traces of fEPSPs at baseline (line) and after low frequency stimulation (dotted line) of acute hippocampal slices of adult rats previously injected with either knockdown AAV5-shRNA#1 (right hemisphere) or AAV5-scrambled#1 (left hemisphere) (n = 6 animals). (Bottom) Representative traces and bar chart of changes in fEPSPs. Baseline fEPSPs were recorded from the apical dendritic layer of CA1. LTD was induced by low frequency stimulation (900 stimuli at 1 Hz) of Schaffer collaterals. The traces show no differences in LTD induction or maintenance between Shrm4-silenced hippocampal slices and scrambled controls.

(**d**) Representative images (top) and histogram (bottom) of the mean intensity of PSD-95 positive puncta (excitatory synapses) immunolabeled in the CA1 region of the hippocampus of rats previously injected with either knockdown AAV5-shRNA#1 (right hemisphere) or AAV5-scrambled#1 (left hemisphere). The results show no differences in PSD95 mean intensity between Shrm4-silenced hippocampal slices and scrambled slices (n= 3 animals).

Scale bar: 20 µm (**e**) (Left) Representative traces and (Right) histograms of amplitude, decay time, area and frequency of mIPSCs recorded from DGGCs of adult rats previously injected with either knockdown AAV5-shRNA#1 (right hemisphere) or AAV5-scrambled#1 (left hemisphere) (n= 3 animals). Amplitude, decay time, area and frequency of mIPSCs do not differ significantly between Shrm4-silenced hippocampal slices and scrambled controls. mIPSCs were measured in the presence of kynurenic acid (3 mM) and lidocaine (500 µM).

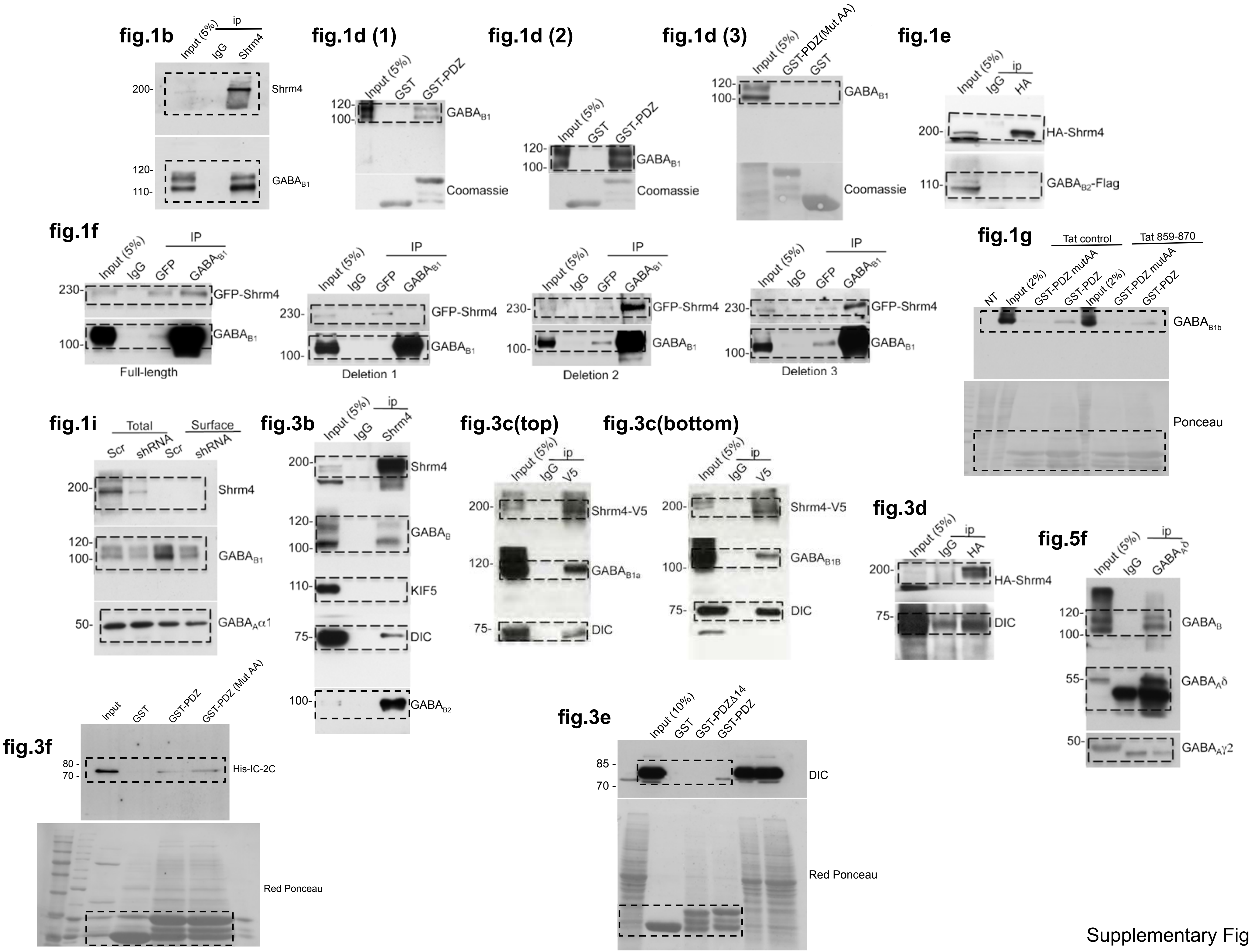

Supplementary Figure 11

Supplementary figure 11. Full versions of the western blots presented in the study. The blots have been labelled with figure panels where they have appeared.

Supplementary Table 1: Distribution of gold nanoparticles in cultured rat cortical neurons immunolabeled with (Shrm4) or without (Control) primary antibodies against Shrm4

Compartments	**Observed Gold, No**	**Observed Points (P)**	**LD Values ($No/P*a_p$)**	**Expected Gold, Ne**	**RLI Values (No/Ne)**	**X^2 Values**
**<u>Shrm4</u>**						
Synaptic bouton	15	5	3	2.66	5.64	57.30*
Post-synaptic terminal	1	1	1	0.53	1.88	0.41
Thin dendrites	102	86	1.19	45.72	2.23	69.27*
Other un-determined structures	4	70	0.06	37.22	0.11	29.65**§**
Cell structure absent	4	75	0.05	39.87	0.10	32.27**§**
Total	126	237	0.53	126	1	188.91**$**
**<u>Control</u>**						
Synaptic bouton	1	1	1	0.60	1.67	0.27
Post-synaptic terminal	0	1	0	0.60	0	0.60
Thin dendrites	17	22	0.77	13.14	1.29	1.13
Other un-determined structures	18	29	0.62	17.32	1.04	0.03
Cell structure absent	10	24	0.42	14.34	0.70	1.31
Total	46	77	0.60	46	1	3.34

Supplementary Table 2: Statistical analysis parameters for Fig. 2

Figure panel	Statistical test	n	Conditions	p value	F
2c (Left) PSD95	Ordinary one-way ANOVA	15, 14, 11	Scrambled, shRNA#1, rescue	p=0,0011	F(2,32)=8,191
2c (Left) Bassoon	Ordinary one-way ANOVA	10, 10, 15	Scrambled, shRNA#1, rescue	p=0,0002	F(2,32)=11.16
2c (Left) GluA2	Ordinary one-way ANOVA	10, 10, 15	Scrambled, shRNA#1, rescue	p<0,0001	F(2,32)=32.72
2c (Left) Synapsin	Ordinary one-way ANOVA	10, 10, 15	Scrambled, shRNA#1, rescue	p=0,0002	F(2,32)=11.22
2c (Right) PSD95	Ordinary one-way ANOVA	15, 14, 11	Scrambled, shRNA#1, rescue	p=0,0011	F(2,37)=2,37
2c (Right) Bassoon	Ordinary one-way ANOVA	15, 20, 15	Scrambled, shRNA#1, rescue	p=0,0148	F(2,32)=4,615
2c (Right) GluA2	Ordinary one-way ANOVA	17, 20, 15	Scrambled, shRNA#1, rescue	p=0,0069	F(2,32)=5,592
2c (Right) Synapsin	Ordinary one-way ANOVA	10, 10, 15	Scrambled, shRNA#1, rescue	p=0,0174	F(2,32)=5,592
2d	Ordinary one-way ANOVA	15, 15, 20	Scrambled, shRNA#1, rescue	p=0,0029	F(2,47)=6,641
2f	Ordinary one-way ANOVA	15, 15, 20	Scrambled, shRNA#1, rescue	p<0,0001	F(2,42)=75.59
2g	Ordinary one-way ANOVA	15, 15, 20	Scrambled, shRNA#1, rescue	p<0,0001	F(2,47)=28.23
